# Seasonal Dynamics of Fruit Flies (Diptera: Drosophilidae) in Forests of the European Russia

**DOI:** 10.3390/insects13080751

**Published:** 2022-08-20

**Authors:** Nikolai G. Gornostaev, Alexander B. Ruchin, Mikhail N. Esin, Aleksei M. Kulikov

**Affiliations:** 1 N.K. Koltsov Institute of Developmental Biology RAS, 119334 Moscow, Russia; 2 Joint Directorate of the Mordovia State Nature Reserve and National Park “Smolny”, 430005 Saransk, Russia

**Keywords:** Drosophilidae, seasonal dynamics, Republic of Mordovia, fauna

## Abstract

**Simple Summary:**

It is the first investigation of drosophilid seasonal population changes considering their biotope association, abundance and species diversity in European Russia. The material was collected using beer traps in different forest biotopes. Beer is an attractive component for fruit flies. Two species were most common (*Drosophila obscura* and *Drosophila* *histrio*). We found three groups of mass species with a significant correlation of seasonal dynamics.

**Abstract:**

(1) Background: Seasonal dynamics of the abundance and species diversity of various insect groups is of great importance for understanding their life cycles; (2) Methods: In our study, Drosophilidae species and their seasonal changes in Mordovia State Nature Reserve were explored. We collected the flies by crown fermental traps in five types of forests (birch, aspen, linden, pine and oak) since May to October in 2019. (3) Results: A total of 4725 individuals belonging to 9 genera and 30 species of drosophilid flies were identified, among them 15 species in 3 genera are new to the Republic of Mordovia. *Drosophila obscura* and *D. histrio* were the most abundant species in traps, the other mass species are *D. kuntzei, D. testacea, D. phalerata, S. rufifrons, D. bifasciata, A. semivirgo,* and *L. quinquemaculata*. (4) Conclusions: We found three groups of mass species with significant correlation of seasonal dynamics, e.g., D.obscura and *D. bifasciata; D. histrio, D. kuntzei, D. phalerata,* and *D. testacea*, and, finally, *A. semivirgo* and *S. rufifrons*. Apparently, the similarity observed in the seasonal dynamics of these drosophilid species is influenced at a high degree by their food preferences and rearing sites.

## 1. Introduction

Most aspects of body physiology, metabolism, and behavior are controlled by the clock and lead to daily or seasonal strategies. The relationship between the timing of life cycle events and seasonal climatic changes (i.e., phenology) is a fundamental biological process in natural systems. Phenology is the main factor determining population dynamics, species interaction, animal movement, and the evolution of life history [1,2]. The timing of phenological events is gradually changing as a result of climate change [3,4,5]. Along with other adaptive mechanisms, plasticity in phenology is essential for maintaining many aspects of biodiversity in a changing environment, such as species demography, species interaction, and species distribution [6,7,8]. In response to seasonal natural changes, the species composition of populations and the number of species in them undergoes significant fluctuations [9,10,11].

The rhythms of the vital activity of insects as poikilothermic animals are also adapted to seasonal environmental changes. Insects are particularly sensitive to an increase or decrease in temperatures above or below their optimum, to frost and drought, as well as to a decrease in the availability of resources, particularly food [4,12]. Therefore, seasonal rhythms of insect activity depend on a variety of environmental factors, most often on temperature, photoperiod, and humidity [10,13,14]. In this regard, the observed climate changes lead to clear shifts in the phenology of species, changes in their life cycles of development and reproduction [15,16,17,18,19,20]. Thus, the seasonal aspects of insect biology are key processes that can link climate change to population conservation and possibly to community composition [21].

Especially clear seasonal rhythms were found in a wide variety of insect groups living in temperate latitudes. In particular, the seasonal activity of species of Carabidae [22,23], Staphylinidae [24], Mordellidae [25], Scarabaeidae [26], Cerambycidae [27], Elateridae [28], and many others. The phenological features of Lepidoptera and Hymenoptera of temperate climate have been well studied [29,30,31,32]. No less interesting are the seasonal dynamics of individual families and species of Diptera. In the forest zone of Russia in the second half of September, there was a gradual increase in the number of Diptera with a peak in mid-October. The autumn increase in the number of Diptera in different biotopes exceeded the summer peak several times [33]. Based on the analysis of the activity of 194 Syrphidae species, ten phenological groups were identified, which differed in peaks of activity during the season [34]. Many parasitic Diptera species depend on the seasonal activity of their hosts, which serve for the development of larvae [35]. Anisopodidae activity occurs at the end of August and autumn [36]. The seasonal activity of *Stomoxys calcitrans* shows one large peak at the end of summer and a second smaller peak just before the end of the flight season [37]. The phenological phases of *Ceratitis capitata* development depended on the abundance of food items—various fruits [38]. Generally known as fruit flies, family Drosophilidae consists of approximately 4000 species worldwide [39,40]. The majority of adult drosophilids feed on the bacteria and yeasts arising from the fermentation of various plant substrates (fruit, tree sap, rotting leaves, etc.). Their larvae also prefer the bacteria and yeasts arising from the fermentation of carbohydrates [41] but some species feed on living mushrooms, living plant tissues as miners, etc., [42]. The aim of the research was to study the species diversity and seasonal dynamics of drosophilids in various forest biotopes of the center of the European part of Russia. The objectives of the research were: (1) study of the species diversity of drosophilids in various biotopes using beer traps; (2) study of the seasonal dynamics of mass species of drosophilids.

## 2. Materials and Methods

### 2.1. Study Area

The study was carried out in the Mordovia State Nature Reserve (European Russia), located on the southern boundary of the taiga zone (54°42′–54°56′ N, 43°04′–43°36′ E; up to 190 m a.s.l.). The Mordovia State Nature Reserve contains natural ecosystems in the center of European Russia, acknowledged as a hotspot for biodiversity [43,44]. The total area of the protected area is 321.62 km^2^ with forest communities covering 89.3% of this area. Pine (*Pinus sylvestris* L.) is the main forest tree species where it forms pure or mixed forest communities. Most of these places are artificial pine plantings of different ages. Birch (*Betula pendula* Roth) is the second commonest tree species and forms predominantly secondary forest communities on old logging or burnt areas. In mixed forests, birch is the main component of the second tier of the forest. Small-leaved linden (*Tilia cordata* Mill.) forms pure stands in the northern part of the Mordovia State Nature Reserve, as well as being important in the development of an undergrowth layer in pine stands and mixed forests. Oak (*Quercus robur* L.) forests occupy relatively small areas mainly on the floodplain of the Moksha River in the western part of the Mordovia State Nature Reserve. Sections of oak forests have also been preserved along the shores of some lakes in the southwestern part of the protected area. The mean annual precipitation is 406.6–681.3 mm. The reserve is located in a temperate zone with a predominance of forest-steppe type of climate. The average annual air temperature ranges from 3.5 to 4.0 °C. The average temperature of the coldest month (January) varies between −11.5–−12.3 °C; temperature drops to −47 °C are noted. The average temperature of the warmest month—July—18.9–19.8 °C. Extreme temperatures in summer reach 37 °C. We collected the flies from May to October in 2019 when average day temperatures allowed insects to be active.

### 2.2. Sampling

Each trap was a plastic 5 L container with a window cut out in it on one side at a distance of 10 cm from the bottom [45]. Two traps were installed in each biotope at a distance of 5 m from each other. The traps were suspended on tree trunks in the crown at a height of 7–8 m. Fermented liquid (beer with added sugar) was used as a luring liquid. The fermentation period of the liquid was one day. The sampling period ranged from 6 to 17 days. All studies in biotopes were carried out by A.B. Ruchin.

The definition of the flies was performed by N.G. Gornostaev with the use of drosophilid key [46]. The systematics of Drosophilidae is interpreted by Grimaldi [47]. Species new to the region are marked with an asterisk “*”.

### 2.3. Statistical Analysis

To estimate correlated changes in the number of species by months and biotopes, Spearman rank order correlations were used according to the percentage of the number of each species in the sample obtained for this species for the entire period of accounting in this biotope. Estimates were obtained using the Statistica 12 program [48]. Diagrams of seasonal dynamics of species are constructed in the Excel program according to the corresponding values of the percentage of the number of this species from the total number in this biotope for the entire accounting season.

To compare the drosophilid fauna in five biotopes, we calculated the Shannon–Weaver biodiversity index and the Simpson dominance index (based on the data in Appendix A).

## 3. Results

### 3.1. Faunistic Composition

Until recently, the fauna of the Drosophilidae of the Republic of Mordovia was totally unknown. The first paper with a short regional drosophilid faunistic list considered ecological questions of insect post-fire forest recovery [49]. This preliminary faunistic list includes 15 species in 6 genera of Drosophildae. Here we give an addition with a new list of Drosophilidae of the Republic of Mordovia consisting of 30 species in 9 genera.

Among the flies collected in beer traps we found 4 genera and 9 species of subfamily Steganinae and 5 genera and 21 species of subfamily Drosophilinae:

Steganinae

1. *Amiota (Amiota) albilabris* (Roth in Zetterstedt, 1860)
2. *Amiota (Amiota) alboguttata* (Wahlberg, 1839)
3. *Amiota (Amiota) rufescens* (Oldenberg, 1914)
*Amiota (Amiota) subtusradiata Duda, 1934
5. *Amiota (Phortica) semivirgo* Maca, 1977
6. *Gitona distigma* Meigen, 1830
7. *Leucophenga maculata* (Dufour, 1839)
8. *Leucophenga quinquemaculata* Strobl, 1893
*Stegana (Steganina) coleoptrata (Scopoli, 1763)

Drosophilinae

10. *Chymomyza amoena (Loew, 1862)
11. *Chymomyza caudatula Oldenberg, 1914
12. *Chymomyza costata* (Zetterstedt, 1838)
13. *Chymomyza fuscimana (Zetterstedt, 1838)
14. *Drosophila (Dorsilopha) busckii Coquillett, 1901
15. *Drosophila (Drosophila) funebris (Fabricius, 1787)
16. *Drosophila (Drosophila) histrio* Meigen, 1830
17. *Drosophila (Drosophila) hydei Sturtevant, 1921
18. *Drosophila (Drosophila) immigrans Sturtevant, 1921
19. *Drosophila (Drosophila) kuntzei Duda, 1924
20. *Drosophila (Drosophila) phalerata* Meigen, 1830
21. *Drosophila (Drosophila) testacea *von Roser, 1840
22. *Drosophila (Drosophila) transversa *Fallen, 1823
23. *Drosophila (Sophophora) bifasciata *Pomini, 1940
24. *Drosophila (Sophophora) melanogaster Meigen, 1830
25. *Drosophila (Sophophora) obscura *Fallen, 1823
26. *Drosophila (Sophophora) tristis Fallen, 1823
27. *Hirtodrosophila confusa (Staeger, 1844)
28. *Hirtodrosophila trivittata (Strobl, 1893)
29. *Scaptodrosophila rufifrons *(Loew, 1873)
30. *Scaptomyza (Hemiscaptomyza) unipunctum (Zetterstedt, 1847)

### 3.2. Seasonal Dynamics of Drosophilidae

As a result of the study, 4725 individuals from 9 genera and 30 species were detected in 2019 (Table 1).

As we can conclude from our results, nine drosophild species (*D. obscura, D. histrio, D. kuntzei, D. testacea, D. phalerata, S. rufifrons, D. bifasciata, A. semivirgo* and *L. quinquemaculata*) were the most abundant in 2019, e.g., each of them with total number of flies caught in traps more than 100 exemplars. The amount of flies belonging to these 9 species is 4496 exemplars, which is 95.15% of total drosophilid number in our collection. We consider the other 21 species collected in amounts less than 100 flies as relatively rare or weakly attracted to this type of traps.

Interestingly, the most abundant species of Drosophilidae demonstrate different patterns of seasonal dynamics. Six species, e.g., *D. obscura, D. histrio, D. kuntzei, D. testacea, D. phalerata,* and *D. bifasciata*, show very strong increases in collected drosophilid numbers in October. However, among this group, *D. obscura* and *D. bifasciata* show additional moderate summer increases in July, and *D. histrio* in May, August, and September. On the contrary, two species, *S. rufifrons* and *A. semivirgo*, show low numbers in May–June increasing in July up to maximum values in August followed by decreases in September–October. One species, *L. quinquemaculata*, demonstrates similar maximal numbers in May and October, decreases in June, noticeable increases in July, and minimal equal numbers in August–September.

### 3.3. Species diversity of Drosophildae

**Figure 1:**
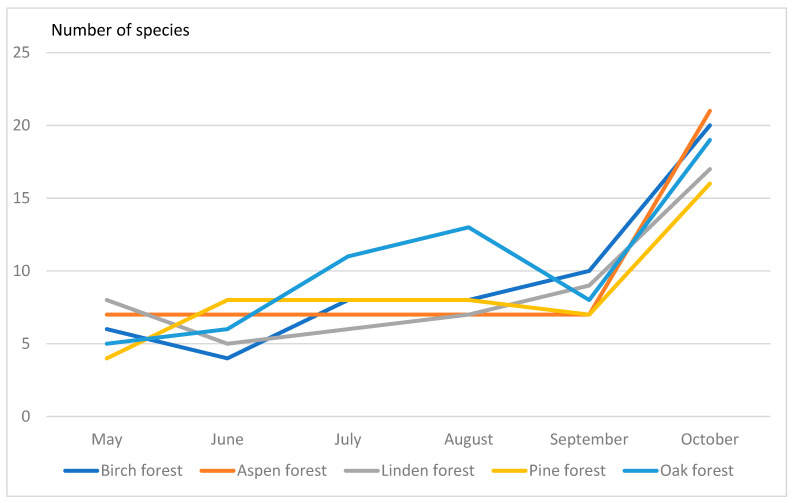
Seasonal changes of species diversity of Drosophildae.

The drosophilid species diversity, e.g., number of collected species, varied between different types of forest since May to October (Figure 1). We found that species diversity have maximal values in October in all types of forest examined.

In birch and linden forests, the number of drosophilid species was 20, in pine—19 species, in aspen—21 species. The greatest species diversity was observed in oak forest (23 species). At the same time, the calculated indices showed interesting results. Thus, according to the Shannon–Weaver index, the most diverse communities were in the linden forest (index 2.11), and the least diverse in the oak forest (index 1.87). In other communities, this index was intermediate and very similar (1.95–1.99). The Simpson index showed that the dominance of one or two drosophilid species is maximal in the oak forest (0.31). At the same time, in the linden forest, the dominance of species is the least pronounced (0.15), i.e., here the community is more aligned (Table A10).

### 3.4. Seasonal Dynamics of Drosophilidae in Five Biotopes

We studied seasonal dynamics of Drosophilidae in five types of forest. We found that the drosophilid abundance was as follows: maximum value was in birch forest (1322) and the lowest in oak forest (640). Interestingly, the number of females exceeded the number of males in traps in all types of forest.

**Figure 2:**
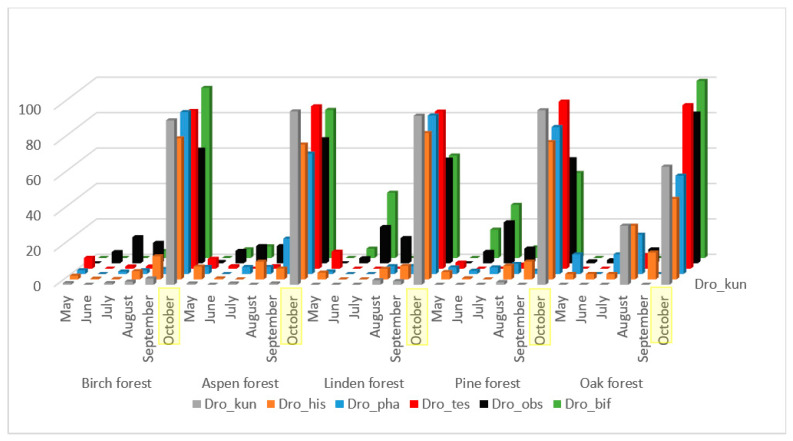
Dynamics of changes in the number of species *D. obcura, D. bifasciata, D. histrio, D. kuntzei, D. phalerata, D. testacea* from May to October as a percentage of the total number for the entire period of accounting in biotope.

The majority of the mass species presented in Figure 2, with the exception of *L. quinquemaculata*, have a significant correlation of population fluctuations throughout the entire accounting season, from May to October (Table 2). These species are characterized by low representation in June, an increase in numbers in July–September, and maximum representation in October. Some differences in seasonal dynamics by biotopes are caused by a small intermediate peak in the abundance of *D. kuntzei, D. histrio*, and *D. phalerata* species in August in oak forest collections, and in *D. obscura* and *D. bifasciata* species in linden and pine forests.

We found the highest significant correlation of seasonal dynamics between closely related species *D. obscura* and *D. bifasciata* (Table 2). They are typical xylosaprobionts, their larvae live mainly in the tissues under the bark and in the fermenting tree sap [42]. The second group with high significant correlation of seasonal dynamics consists of *D. histrio, D. kuntzei, D. phalerata*, and *D. testacea*. All these species are mycetobionts, their larvae live in various fungi.

**Figure 3:**
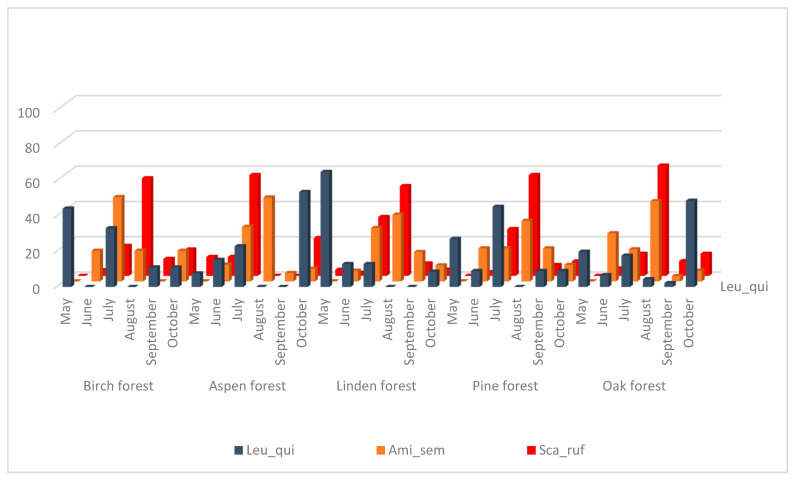
Dynamics of changes in the number of species *L. quinquemaculata, A. semivirgo, S. rufifrons* from May to October as a percentage of the total number for the entire period of accounting in biotope.

The species *Amiota semivirgo* and *S. rufifrons* also have significantly correlated seasonal dynamics (Figure 3, Table 3) but their main peak is observed in July–August in all biotopes, and in September–October, the number of collected flies decreases sharply. These species are also xylosaprobionts. Seasonal fluctuations in the number of *L. quinquemaculata* species do not show a significant correlation with any of drosophilid species and show a maximum in May and July in birch and pine forests, in May only in the linden forest, and in October in aspen and oak forests. The larvae of *L. quinquemaculata* could be found mainly in bracket fungi so they occupy a rather separate and specific ecological niche.

The group of mycetobionts developing mainly in various species of basidiomycetes includes mass species *D. histrio, D. kuntzei, D. phalerata, D. testacea* (Table A2, Table A3, Table A4 and Table A5), and *L. quinquemaculata* rearing in bracket fungi (Table A9), which were found in an amount of more than 100 specimens (Table 4).

The second large ecological group of drosophila includes xylosaprobionts (*D. obscura, D. bifasciata, S. rufifrons, A. semivirgo*) (Table A1, Table A6, Table A7 and Table A8); their larvae live mainly in tissues under the bark and in fermenting tree sap (Table 5).

## 4. Discussion

The influence of seasonal changes on the abundance of Drosophilidae has been studied mainly in tropical and temperate climatic zones. Their abundance in tropical regions is affected by precipitation, and in regions with a temperate climate, temperature fluctuations are most affected [49,50,51,52,53]. Our studies have shown that Drosophilidae in central Russia have one peak in numbers, which begins at the end of September with a maximum in mid-October. At this time, daytime temperatures were recorded at no higher than 15 °C, and at night—no more than 10 °C. At the same time, throughout the season, the number of this family in traps was more or less constant without sharp peaks or lows. Similar dynamics were found in experiments in Uşak province, Turkey [54]. The average temperature of October and November with the highest numbers of Drosophilidae was from 5 to 10 °C. At the same time, in September, when the temperature was more favorable for fruit flies, the amount of catch was less [54].

Our work is the first study considering seasonal dynamics of Drosophilidae in European Russia. A total of 4725 individuals belonging to 9 genera and 30 species of drosophilid flies were identified in Mordovia State Nature Reserve. *D. obscura* and *D. histrio* were the most abundant species in beer traps. At the same time, seven more species (*D. bifasciata, D. kuntzei, D. phalerata, D. testacea, L. quinquemaculata*) were observed in traps with high numbers.

Among the 30 species of drosophila collected in Republic of Mordovia, 5 species of the genus *Drosophila* (*D. busckii, D. funebris, D. hydei, D. immigrans, D. melanogaster*) are synanthropic, i.e., closely related to humans and their activities. They live and breed in places where they can find fermenting and rotting fruits and vegetables, wine, beer and juices [42,55,56,57,58]. These species occur in small numbers in wild biotopes, apparently, due to migration attempts or wind transport. Most of the other drosophilid species (24 in our collections) are typical forest dwellers, which rarely occur far from the forest or groups of trees. The larvae of these drosophilids develop in moist tissues under the bark of deciduous trees, in fermenting tree sap, and in various fungi, including ascomycetes and tinders [59,60,61]. The larvae of the last species in our faunistic list, *Gitona distigma* Mg., according to the literature, are phytophages living in inflorescences of family Asteraceae plants, e.g., *Sonchus* and *Crepis* species [42]. Therefore, *G. distigma* may occur in different biotopes, not only in forests, sometimes even in people’s houses.

Here we compare the drosophilid fauna of the Republic of Mordovia with other regions of European Russia, we used data for the Moscow region—35 species [62], Voronezh region—18 species [62,63], Samara region—13 species [62,64], and North Karelia—19 species [65] (Table 6).

As can be seen from Table 6, the largest number of drosophilid species was observed in the Moscow region and Republic of Mordovia; this is a consequence of the special studies of this family conducted in these regions. Nevertheless, by now the degree of similarity is about 2/3 of the total number of species, we have found 21 common species for the fauna of the Republic of Mordovia and the Moscow region.

We studied seasonal dynamics of Drosophilidae in five types of forest (birch, aspen, linden, pine, and oak). Interestingly, the highest abundance of drosophilids was found in October in all types of the forests examined. We found that the drosophilid abundance demonstrated maximum value in birch forest and the lowest value in oak forest. In our collection we found representatives of two main ecological groups—mycetobionts and xylosaprobionts.

The total number of mass mycetobionts (2493) is 55.45% of the total number of drosophilid mass species (4496) and 52.76% of the total number of collected flies. At the same time, the larvae of *D. histrio, D. kuntzei, D. phalerata* and *D. testacea* develop mainly in the fruit bodies of basidiomycetes, and the larvae of *L. quinquemaculata* develop in the bracket fungi. As can be seen from Table 4, the number of imagos of *D. histrio, D. kuntzei, D. phalerata* and *D.testacea* collected in the oak forest is minimal, and several times less than in other biotopes. On the contrary, *L. quinquemaculata* imagos were collected in maximum quantity in the oak forest. We suggest that this is due to noticeable differences in the composition of the mycoflora of oak forests and other types of forests. Apparently, the number of basidiomycetes growing in the oak forest was minimal or their species composition was less attractive for these drosophilid mycetobionts (*D. histrio, D. kuntzei, D. phalerata,* and *D. testacea*). On the contrary, bracket fungi, apparently, occur most often in the oak forest, which explains the largest number of *L.quinquemaculata* collected here. The question of the relationship of various drosophilid species with fungi in Republic of Mordovia has not been studied yet but perhaps deserves a separate investigation.

The total number of mass xylosaprobionts (2003) is 44.55% of the total number of drosophilid mass species (4496) and 42.39% of the total number of flies collected. As can be seen from Table 5, xylosaprobionts demonstrate the maximum abundance in oak and birch forests. Apparently, this is due to the greatest number of wounds on tree trunks in these biotopes, which attract drosophilids of these species (*D. obscura, D. bifasciata, S. rufifrons, A. semivirgo*).

We found the highest significant correlation of seasonal dynamics between closely related xylosaprobiont species *D. obscura* and *D. bifasciata.* The second group with high significant correlation of seasonal dynamics consists of mycetobiont species *D. histrio, D. kuntzei, D. phalerata,* and *D. testacea*. The third group includes xylosaprobiont species *A. semivirgo* and *S. rufifrons*. Apparently, the similarity observed in the seasonal dynamics of some drosophilid species is influenced at a high degree by their food preferences and rearing sites.

We also analyzed species communities in five biotopes by calculating the Shannon–Weaver index and the Simpson index. It turned out that the greatest differences were found between oak and linden forests: the most diverse species community lives in the linden forest and the least diverse in the oak forest. On the contrary, the dominance of drosophilid species in the linden forest is the least pronounced, and in the oak forest it is the largest among all biotopes (Table A10).

In addition, according to our data, the mass species of drosophilids of the Republic of Mordovia show a different picture of seasonal population peaks. They can be divided into different types: species with summer–autumn peaks of abundance (*D. obscura* and *D. bifasciata*), with spring–autumn peaks (*D. histrio, D. testacea*), only with summer peaks (*A. semivirgo* and *S. rufifrons*), only with autumn peaks (*D. kuntzei, D. phalerata*), and with three peaks of abundance (*L. quinquemaculata*) (Table 7). Therefore, we can conclude that the presence of two or three peaks in numbers of abundance suggests the presence of two or three generations in these drosophilids. However, the presence of one peak number in our collections does not negate the possibility of having two generations, for example, in *D. kuntzei* and *D. phalerata*. Perhaps, for these mycetobiont species, beer traps become less attractive in the summer during the mushroom abundance season. Interestingly, for six mass species of drosophilids, the autumn peak of abundance is the maximum.

## 5. Conclusions

In our study, Drosophilidae species and their seasonal changes in Mordovia State Reserve were explored. It is the first investigation of drosophilid seasonal population changes considering their biotope association, abundance and species diversity in European Russia. We collected the flies by crown fermental traps in five types of forests (birch, aspen, linden, pine, and oak) from May to October in 2019. A total of 4725 individuals belonging to 9 genera and 30 species of drosophilid flies were identified, among them 15 species in 3 genera are new to Republic of Mordovia. *Drosophila obscura* Fll. and *D. histrio* Mg. were the most abundant species in traps, the other mass species are *D. kuntzei, D. testacea, D. phalerata, S. rufifrons, D. bifasciata, A. semivirgo,* and *L. quinquemaculata.* Interestingly, the highest abundance of drosophilids and their species diversity was found in October in all types of the forests examined. We found the highest significant correlation of seasonal dynamics between closely related species *D. obscura* and *D. bifasciata,* the second group with high significant correlation of seasonal dynamics consists of *D. histrio, D. kuntzei, D. phalerata,* and *D. testacea,* and finally the third group consists of *A. semivirgo* and *S. rufifrons.* Apparently, the similarity observed in the seasonal dynamics of these drosophilid species is influenced at high degree by their food preferences and rearing sites.

## Figures and Tables

**Table 1:** Number of Drosophilidae flies collected in traps.

Species	May	June	July	August	September	October	Total Amount
*Amiota albilabris*	0	0	3	2	0	1	6
*Amiota alboguttata*	0	0	1	1	0	5	7
*Amiota rufescens*	0	0	3	1	0	0	4
*Amiota semivirgo*	0	27	56	75	20	18	196
*Amiota subtusradiata*	0	0	1	2	1	0	4
*Gitona distigma*	0	1	0	0	1	25	27
*Leucophenga maculata*	0	0	1	0	0	17	18
*Leucophenga quinquemaculata*	32	9	22	3	3	33	102
*Stegana coleoptrata*	0	1	1	0	0	0	2
*Chymomyza amoena*	0	0	0	0	0	15	15
*Chymomyza caudatula*	1	0	0	0	0	0	1
*Chymomyza costata*	0	0	0	0	0	1	1
*Chymomyza fuscimana*	0	0	0	0	0	1	1
*Drosophila bifasciata*	0	12	26	9	0	216	263
*Drosophila busckii*	0	0	0	0	0	3	3
*Drosophila funebris*	0	0	0	1	0	12	13
*Drosophila histrio*	45	6	2	87	126	945	1211
*Drosophila hydei*	0	0	1	0	0	5	6
*Drosophila immigrans*	0	0	0	0	12	51	63
*Drosophila kuntzei*	2	0	2	9	9	495	517
*Drosophila melanogaster*	0	0	0	0	0	6	6
*Drosophila obscura*	6	60	167	129	34	881	1277
*Drosophila phalerata*	7	1	6	12	13	254	293
*Drosophila testacea*	24	1	1	4	4	336	370
*Drosophila transversa*	0	0	0	0	3	20	23
*Drosophila tristis*	0	0	0	0	0	1	1
*Hirtodrosophila confusa*	13	0	0	1	2	8	24
*Hirtodrosophila trivittata*	0	0	0	0	0	3	3
*Scaptodrosophila rufifrons*	5	9	67	135	22	29	267
*Scaptomyza unipunctum*	0	0	0	0	0	1	1
Total	135	127	360	471	250	3382	4725

**Table 2:** Correlation of seasonal dynamics of the number of species *D.obcura, D.bifasciata, D. histrio, D. kuntzei, D. phalerata, D. testacea, L. quinquemaculata* from May to October in five forest biotopes (Spearman rank order correlations). Significant correlation coefficients are highlighted in red, with values greater than 0.6 in bold.

Variable	Dro_bif	Dro_his	Dro_kun	Dro_obs	Dro_pha	Dro_tes	Leu_qui
Dro_bif	1.000	0.187	0.355	**0.778**	0.353	0.335	0.239
Dro_his	0.187	1.000	**0.667**	0.263	**0.663**	**0.657**	−0.256
Dro_kun	0.355	**0.667**	1.000	0.558	**0.709**	**0.698**	−0.075
Dro_obs	**0.778**	0.263	0.558	1.000	0.439	0.275	0.040
Dro_pha	0.353	**0.663**	**0.709**	0.439	1.000	0.542	−0.014
Dro_tes	0.335	**0.657**	**0.698**	0.275	0.542	1.000	0.241
Leu_qui	0.239	−0.256	−0.075	0.040	−0.014	0.241	1.000

**Table 3:** Correlation of seasonal dynamics of the number of species *L. quinquemaculata, A. semivirgo, S. rufifrons* from May to October in five forest biotopes (Spearman’s rank order correlations). Significant correlation coefficients are highlighted in red, with values greater than 0.6 in bold.

Variable	Ami_sem	Leu_qui	Sca_ruf
Ami_sem	1.000	−0.308	**0.655**
Leu_qui	−0.308	1.000	−0.221
Sca_ruf	**0.655**	−0.221	1.000

**Table 4:** Total collected specimens of mycetobiont drosophilid species in five biotopes.

Biotopes	*D. histrio*	*D. kuntzei*	*D. phalerata*	*D. testacea*	*L. quinquemaculata*	Total
Birch forest	476	111	140	82	9	818
Aspen forest	180	140	25	71	14	430
Linden forest	184	196	67	115	23	585
Pine forest	338	67	53	88	11	557
Oak forest	33	3	8	14	45	103
Total	1211	517	293	370	102	2493

**Table 5:** Total collected specimens of xylosaprobiont drosophilid species in five biotopes.

Biotopes	*D. obscura*	*S. rufifrons*	*D. bifasciata*	*A. semivirgo*	Total
Birch forest	251	94	76	23	444
Aspen forest	257	43	61	42	403
Linden forest	210	57	19	66	352
Pine forest	216	49	50	32	347
Oak forest	343	24	57	33	457
Total	1277	267	263	196	2003

**Table 6:** Comparison of drosophilid fauna in five regions of European Russia.

Species	Republic of Mordovia	Moscow Region	Samara Region	Voronezh Region	North Karelia
*Amiota albilabris*	+	−	−	−	−
*Amiota alboguttata*	+	+	−	−	−
*Amiota rufescens*	***+***	−	−	−	−
*Amiota semivirgo*	+	−	+	+	−
*Amiota subtusradiata*	+	+	−	−	−
*Amiota variegata*	−	−	+	+	−
*Gitona distigma*	+	−	+	+	−
*Leucophenga maculata*	+	−	−	−	−
*Leucophenga quinquemaculata*	+	+	−	−	−
*Stegana coleoptrata*	+	+	−	−	−
*Stegana furta*	−	+	+	−	+
*Stegana hypoleuca*	−	+	−	−	−
*Stegana mehadiae*	−	+	−	−	−
*Stegana similis*	−	+	−	−	−
*Chymomyza amoena*	+	+	−	+	−
*Chymomyza caudatula*	+	+	−	−	−
*Chymomyza costata*	+	+	−	−	+
*Chymomyza distincta*	−	+	−	−	−
*Chymomyza fuscimana*	+	+	−	−	−
*Drosophila alpina*	−	−	−	−	+
*Drosophila bifasciata*	+	+	−	−	+
*Drosophila busckii*	+	+	+	−	+
*Drosophila funebris*	+	+	+	+	+
*Drosophila histrio*	+	+	−	+	+
*Drosophila hydei*	+	−	−	+	−
*Drosophila immigrans*	+	+	−	−	−
*Drosophila kuntzei*	+	−	−	−	−
*Drosophila limbata*	−	+	+	+	−
*Drosophila littoralis*	−	+	−	−	+
*Drosophila montana*	−	−	−	−	+
*Drosophila melanogaster*	+	+	+	+	+
*Drosophila obscura*	+	+	−	−	+
*Drosophila phalerata*	+	+	+	+	−
*Drosophila subarctica*	−	−	−	−	+
*Drosophila subsilvestris*	−	+	−	−	+
*Drosophila testacea*	+	+	−	−	+
*Drosophila transversa*	+	+	+	−	+
*Drosophila tristis*	+	−	−	−	−
*Hirtodrosophila cameraria*	−	+	−	+	+
*Hirtodrosophila confusa*	+	+	−	−	−
*Hirtodrosophila toyohiokadai*	−	+	−	−	−
*Hirtodrosophila trivittata*	+	+	+	−	−
*Lordiphosa fenestrarum*	−	−	+	−	−
*Microdrosophila congesta*	−	−	−	−	+
*Scaptodrosophila rufifrons*	+	+	+	+	−
*Scaptomyza consimilis*	−	+	−	+	−
*Scaptomyza flava*	−	+	−	+	−
*Scaptomyza graminum*	−	+	−	+	+
*Scaptomyza griseola*	−	−	−	+	−
*Scaptomyza pallida*	−	+	−	+	+
*Scaptomyza unipunctum*	+	−	−	+	0
**Total**	**30**	**35**	**13**	**18**	**19**

**Table 7:** Total month-to-month numbers of Drosophilidae in 2019.

Species	May	June	July	August	September	October	Probable Number of Generations
*Drosophila obscura*	6	60	167	129	34	881	2
*Drosophila histrio*	45	6	2	87	126	945	2
*Drosophila kuntzei*	2	0	2	9	9	495	1
*Drosophila testacea*	24	1	1	4	4	336	2
*Drosophila phalerata*	7	1	6	12	13	254	1
*Scaptodrosophila rufifrons*	5	9	67	135	22	29	1–2
*Drosophila bifasciata*	0	12	26	9	0	216	2
*Amiota semivirgo*	0	27	56	75	20	18	1–2
*Leucophenga quinquemaculata*	32	9	22	3	3	33	2–3

## Data Availability

The data presented in the study are available in the article.

## Appendix A

Here we present the seasonal dynamics for the most abundant (>100 flies collected) drosophilid species for every type of forest (Table A1, Table A2, Table A3, Table A4, Table A5, Table A6, Table A7, Table A8 and Table A9) and total numbers of drosophilid specimens collected in five biotopes with calculated Shannon–Weaver and Simpson indexes (Table A10):

**Table A1:** Seasonal dynamics of *Drosophila obscura* in five biotopes.

Biotopes	May	June	July	August	September	October	Total Amount
Birch forest	0	16	37	29	8	161	251
Aspen forest	2	18	25	25	7	180	257
Linden forest	0	6	43	30	8	123	210
Pine forest	0	14	50	18	7	127	216
Oak forest	4	6	12	27	4	290	343
Total amount	6	60	167	129	34	881	1277

**Table A2:** Seasonal dynamics of *Drosophila histrio* in five biotopes.

Biotopes	May	June	July	August	September	October	Total Amount
Birch forest	10	2	1	22	62	379	476
Aspen forest	13	1	0	18	11	137	180
Linden forest	7	0	0	11	14	152	184
Pine forest	14	2	0	26	34	262	338
Oak forest	1	1	1	10	5	15	33
Total amount	45	6	2	87	126	945	1211

**Table A3:** Seasonal dynamics of *Drosophila kuntzei* in five biotopes.

Biotopes	May	June	July	August	September	October	Total Amount
Birch forest	1	0	1	2	4	103	111
Aspen forest	1	0	1	0	1	137	140
Linden forest	0	0	0	5	4	187	196
Pine forest	0	0	0	1	0	66	67
Oak forest	0	0	0	1	0	2	3
Total amount	2	0	2	9	9	495	517

**Table A4:** Seasonal dynamics of *Drosophila testacea* in five biotopes.

Biotopes	May	June	July	August	September	October	Total Amount
Birch forest	5	0	1	1	2	73	82
Aspen forest	4	1	0	0	0	66	71
Linden forest	11	0	0	0	2	102	115
Pine forest	3	0	0	2	0	83	88
Oak forest	1	0	0	1	0	12	14
Total amount	24	1	1	4	4	336	370

**Table A5:** Seasonal dynamics of *Drosophila phalerata* in five biotopes.

Biotopes	May	June	July	August	September	October	Total Amount
Birch forest	3	0	2	3	4	128	140
Aspen forest	1	0	1	1	5	17	25
Linden forest	1	0	0	3	3	60	67
Pine forest	2	1	2	3	1	44	53
Oak forest	0	0	1	2	0	5	8
Total amount	7	1	6	12	13	254	293

**Table A6:** Seasonal dynamics of *Scaptodrosophila rufifrons* in five biotopes.

Biotopes	May	June	July	August	September	October	Total Amount
Birch forest	0	3	16	52	9	14	94
Aspen forest	3	3	16	11	4	6	43
Linden forest	2	1	19	29	4	2	57
Pine forest	0	1	13	28	3	4	49
Oak forest	0	1	3	15	2	3	24
Total amount	5	9	67	135	22	29	267

**Table A7:** Seasonal dynamics of *Drosophila bifasciata* in five biotopes.

Biotopes	May	June	July	August	September	October	Total Amount
Birch forest	0	0	0	3	0	73	76
Aspen forest	0	3	4	3	0	51	61
Linden forest	0	1	7	0	0	11	19
Pine forest	0	8	15	3	0	24	50
Oak forest	0	0	0	0	0	57	57
Total amount	0	12	26	9	0	216	263

**Table A8:** Seasonal dynamics of *Amiota semivirgo* in five biotopes.

Biotopes	May	June	July	August	September	October	Total Amount
Birch forest	0	4	11	4	0	4	23
Aspen forest	0	4	13	20	2	3	42
Linden forest	0	4	20	25	11	6	66
Pine forest	0	6	6	11	6	3	32
Oak forest	0	9	6	15	1	2	33
Total amount	0	27	56	75	20	18	196

**Table A9:** Seasonal dynamics of *Leucophenga quinquemaculata* in five biotopes.

Biotopes	May	June	July	August	September	October	Total Amount
Birch forest	4	0	3	0	1	1	9
Aspen forest	1	2	3	1	0	7	14
Linden forest	15	3	3	0	0	2	23
Pine forest	3	1	5	0	1	1	11
Oak forest	9	3	8	2	1	22	45
Total amount	32	9	22	3	3	33	102

**Table A10:** Drosophilid specimens collected in five biotopes with calculated Shannon–Weaver and Simpson indexes.

	Birch Forest	Aspen Forest	Linden Forest	Pine Forest	Oak Forest
Amiota (Phortica) semivirgo Maca, 1977	23	42	66	32	33
Amiota (Amiota) albilabris (Roth in Zetterstedt, 1860)	0	0	0	0	6
Amiota (Amiota) alboguttata (Wahlberg, 1839)	0	0	0	0	7
Amiota (Amiota) rufescens (Oldenberg, 1914)	0	0	0	2	2
Amiota (Amiota) subtusradiata Duda, 1934	0	0	1	0	3
Gitona distigma Meigen, 1830	1	1	1	1	23
Leucophenga maculata (Dufour, 1839)	1	2	4	1	10
Leucophenga quinquemaculata Strobl, 1893	9	14	23	11	45
Stegana (Steganina) coleoptrata (Scopoli, 1763)	0	0	1	0	1
Chymomyza amoena (Loew, 1862)	4	2	0	3	6
Chymomyza caudatula Oldenberg, 1914	0	0	1	0	0
Chymomyza costata (Zetterstedt, 1838)	0	0	1	0	0
Chymomyza fuscimana (Zetterstedt, 1838)	0	1	0	0	0
Drosophila (Dorsilopha) busckii Coquillett, 1901	1	1	0	1	0
Drosophila (Drosophila) funebris (Fabricius, 1787)	6	1	4	0	2
Drosophila (Drosophila) histrio Meigen, 1830	476	180	184	338	33
Drosophila (Drosophila) hydei Sturtevant, 1921	4	1	0	1	0
Drosophila (Drosophila) immigrans Sturtevant, 1921	23	2	14	13	11
Drosophila (Drosophila) kuntzei Duda, 1924	111	140	196	67	3
Drosophila (Drosophila) phalerata Meigen, 1830	140	25	67	53	8
Drosophila (Drosophila) testacea von Roser, 1840	82	71	115	88	14
Drosophila (Drosophila) transversa Fallen, 1823	8	2	1	10	2
Drosophila (Sophophora) melanogaster Meigen, 1830	1	2	3	0	0
Drosophila (Sophophora) bifasciata Pomini, 1940	76	61	19	50	57
Drosophila (Sophophora) obscura Fallen, 1823	251	257	210	216	343
Drosophila (Sophophora) tristis Fallen, 1823	0	0	0	0	1
Hirtodrosophila confusa (Staeger, 1844)	10	3	3	3	5
Hirtodrosophila trivittata (Strobl, 1893)	1	1	0	1	0
Scaptodrosophila rufifrons (Loew, 1873)	94	43	57	49	24
Scaptomyza (Hemiscaptomyza) unipunctum (Zetterstedt, 1847)	0	0	0	0	1
Shannon–Weaver index	**1.98**	**1.99**	**2.11**	**1.95**	**1.87**
Simpson index	**0.20**	**0.19**	**0.15**	**0.21**	**0.31**
